# Estradiol Alleviates Intervertebral Disc Degeneration through Modulating the Antioxidant Enzymes and Inhibiting Autophagy in the Model of Menopause Rats

**DOI:** 10.1155/2018/7890291

**Published:** 2018-12-23

**Authors:** Lin-Yu Jin, Zhen-Dong Lv, Kun Wang, Lie Qian, Xiao-Xing Song, Xin-Feng Li, Hong-Xing Shen

**Affiliations:** ^1^Department of Spine Surgery, Renji Hospital, Shanghai Jiao Tong University School of Medicine, Shanghai 200240, China; ^2^Department of Anesthesiology, Ruijin Hospital, Shanghai Jiao Tong University School of Medicine, Shanghai, China

## Abstract

**Objective:**

To investigate the effects of menopause on redox balance in the intervertebral disc and to examine whether oxidative stress and autophagy were associated with disc degeneration in menopause rats.

**Methods:**

Thirty female Sprague-Dawley rats were randomly divided into three groups (sham, ovariectomized with vehicle, and ovariectomized with estrogen). At the end of the 3-month treatment, the rats were examined by 3.0 T MRI. Serum estradiol (E2) level was measured. Redox balance of nucleus pulposus was determined by measuring total antioxidant capacity (T-AOC), superoxide dismutase (SOD), catalase (CAT), glutathione peroxidase (GSH-Px), glutathione (GSH), and oxidized glutathione (GSSG). Transmission electron microscopy (TEM), immunohistochemical staining, and Western blot were used to determine the nucleus pulposus autophagy level. At the same time, Spearman's correlation coefficient was used to describe the relationship between intervertebral disc grade, oxidative stress status, serum E2, and autophagy level.

**Results:**

The level of serum E2 was significantly decreased by ovariectomy and can be corrected by the estrogen replacement therapy (ERT). In OVX rats, an increased oxidative stress and high level of autophagy were observed in nucleus pulposus tissue. ERT prevented the intervertebral disc degeneration (IVDD), restored the redox balance, and reduced autophagy level.

**Conclusion:**

Ovariectomy induced oxidative stress, autophagy, and intervertebral disc degeneration. Autophagy of the intervertebral disc was negatively correlated with oxidative stress, and the level of autophagy can be reduced by ERT through modulating the redox balance and downregulating the autophagy level. Regulating the redox balance of IVD may be a potential therapeutic option for degeneration of the disc in the postmenopausal women.

## 1. Introduction

Menopause occurs gradually between the ages of 45 and 55 years during women's life, leading to a significant depression of estrogen level [1]. This process influences many organs and system metabolism in postmenopausal women. Recently, accumulating evidences showed that older women had a higher prevalence and severity of disc degeneration compared with older men [2–5]. An intervertebral disc (IVD) is a fibrocartilaginous tissue and is composed of three distinct but interdependent tissues: annulus fibrosis (AF), nucleus pulposus (NP), and cartilage endplate (EP). IVDs, as a shock absorber system, can transfer loads and weaken energy that are imposed on the skeletal spine [6]. Intervertebral disc degeneration (IVDD) is characterized by reduced water content, depression of proteoglycan synthesis, inappropriate collagen types, and abnormal production of the extracellular matrix (ECM) [7, 8]. Commonly, the NP tissue plays a major role in the functional composition of IVD, and the pathological changes of the NP tissue are important causes of disc degeneration [9]. Degeneration of the intervertebral disc is the main contributor to back pain. The back pain imposes heavy economic burdens on human society [10]. Regrettably, the current therapy strategies, such as conservative treatment and/or surgery, do not keep the normal function of IVD. Therefore, pathogenesis of IVD and remedial methods for IVDD are still the main area in research about IVD. Several articles indicated that 17*β*-estradiol (E2) could promote AF cell and NP cell proliferation by reducing the level of apoptosis in vitro [11–13]. Interestingly, postmenopausal women, receiving estrogen replacement treatment (ERT), can sustain a higher IVD height than untreated menopausal women [14]. For animal intervertebral discs, previous studies have shown that OVX female rats are sensitive to IVDD, and E2 supplementation can retard the progress of pathology of the IVDD [15, 16]. However, although significant advances have been made in order to understand the protective effects of E2 that can reverse or retard the pathogenesis of IVDD, the underlying mechanisms are not well understood.

The process of IVDD induced by estrogen depletion may be partly caused by oxidative stress, which is defined as imbalance between oxidants and antioxidants [17]. Recent studies have reported that the occurrence and development of IVDD are related to oxidative stress [18, 19]. More importantly, postmenopausal women have higher levels of oxidative stress, suggesting that antioxidant capacity may be associated with decreased estrogen [20, 21]. Interestingly, estrogen replacement therapy (ERT) could be able to restore antioxidant status [22]. Autophagy, an important cellular protective mechanism, can respond to different kinds of cellular stress. Furthermore, several in vitro studies suggested that autophagy has an important protective effect on IVD cells [23, 24]. Evidences mainly obtained from the above studies suggest that the oxidative stress induced by the estrogen loss may be a risk factor for the IVDD, and autophagy level may play a protective role with the absence of estrogen. Nevertheless, the relationships between estrogen and redox system/autophagy level have not been addressed so far in intervertebral discs. Therefore, our objective was to investigate the effects of changes in estrogen levels, redox balance, and autophagy on intervertebral discs in ovariectomized rats.

## 2. Methods and Materials

### 2.1. Study Design

The present study included thirty six-week-old female Sprague-Dawley rats, and they were randomized and divided into three groups, which were sham surgery (sham), oophorectomy (OVX), and 10 *μ*g/kg/day 17*β*-estradiol (OVX + E2). Bilateral oophorectomy was performed under anesthesia with pentobarbital for the sham, OVX + veh, and OVX + E2 groups. E2 supplementation was performed for 12 weeks; then, we used the 3.0 T MRI (Siemens Symphony, Erlangen, Germany) to examine the IVD of rats. The IVDD was evaluated by the quantitative T2 mapping in the MRI sagittal plane. Immediately after radiographic examinations, the intervertebral discs and blood samples were acquired from the rats treated with overdose pentobarbital. No rat died during the research before they were treated with euthanasia. All experiments were in accordance with the *Guide for the Care and Use of Laboratory Animals* and have been approved by the Ethics Committee of Renji Hospital.

### 2.2. Magnetic Resonance Imaging Examination

**Figure 1:**
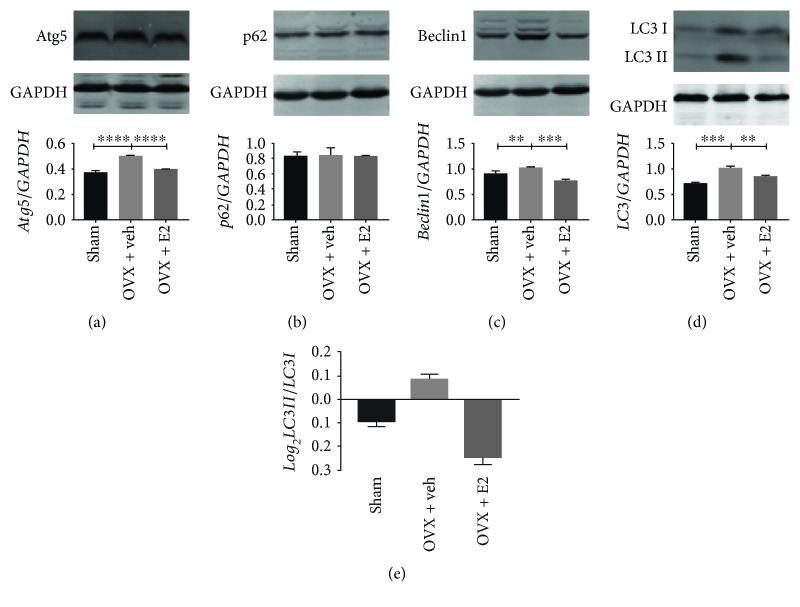
Autophagy level in the NP tissue of OVX was higher than that of the other two groups. An increase in protein levels of Atg5 (a), Beclin1 (c), and LC3-II (d) and the LC3 I/LC3 II ratio (e). However, there are no significant differences in the p62 (b). ^∗∗^*P* < 0.05; ^∗∗∗∗^*P* < 0.01.

T2 mapping magnetic resonance (MR) imaging sequence is a reliable method for monitoring the dipolar interaction of water proton molecule movements in the extracellular matrix of collagen and proteoglycan [25]. Lumbar MRI images of the three groups were obtained with a 3.0 T MR machine (Siemens), covering the L1–L6 IVDs. The same procedure was used to scan the spines: acquisition time of the T2 mapping sequence of L5–6 disc for each spine was approximately 15 min. Then, the regions of interest (ROIs), which were displayed in Figure 1(a), were evaluated by T2 mapping relaxation time; ROI 1 covered the ventral annulus fibrosus (VAF), ROI 2 covered the ventral border zone (BZ), ROI 3 covered the NP, ROI 4 covered the dorsal BZ, and ROI 5 covered the dorsal annulus fibrosus (DAF). The standard ratio of these ROIs was determined based on microstructure evaluation under a microscope: ROI 1 accounted for 25.3% of the disc diameter, ROIs 2 and 4 each were 13.8%, ROI 3 was 35.9%, and ROI 5 measured 11.2%.

### 2.3. Histological Evaluation

4% paraformaldehyde was used to fix the spinal tissues, which were washed twice with phosphate-buffered saline (PBS). 24 hours later, the tissues underwent the procedure of decalcification for one month. Immediately after that, the tissues were embedded in paraffin. Then, the L5–L6 disc segments were sliced into 4 *μ*m thick sections. Obtained in the sagittal plane, it was treated with xylene to remove paraffin, rehydrated in a gradient alcohol bath, and then rinsed 3 times with PBS. Sections were stained with either hematoxylin-eosin (HE), safranin-O (SO) green, or picrosirius rend (PR). The slides were observed with a digital microscope (Olympus, Japan), and a scoring system, according to the previous study [16], was used to evaluate the disc degeneration. These slides were independently and blindly assessed by 2 observers, and the average results of the 2 observers were regarded as the final data.

### 2.4. Immunohistochemistry for Autophagy Level of IVD

The paraffin-embedded L5–L6 segments were cut into 4 *μ*m thickness, and then the slides underwent paraffin removal and were rehydrated in graded alcohol baths. After being treated with antigen retrieval and blocking solution, slides were incubated with an LC-3B monoclonal antibody (1 : 100, CST Inc., USA) at 4°C overnight. Then, the sections were incubated with a secondary antibody dilution (1 : 500, CST Inc., USA) for 30 min. Finally, slides were treated with DAB solution for 5 min after a double wash by PBS. Finished works were observed with a digital microscope (Olympus, Japan). The semiquantitative analysis method was used to evaluate the density of sections by the Image-Pro Plus (IPP) 6.0 software, and the average results were addressed under 400x magnification images.

### 2.5. Measurement of Redox Balance in IVD

The fresh NP tissue of IVD was ground and made into protein homogenate, and it was centrifuged at 3000 rpm for 15 min. An antioxidant enzyme activity test kit (Nanjing Jiancheng Bioengineering Institute, Jiangsu, China) was used to detect the activities of T-AOC, SOD, CAT, and GSH-Px and the level of T-GSH and GSSG. All procedures were performed according to the instruction book of the kit.

### 2.6. Detection of Serum E2

The blood samples after standing at room temperature for 2 hours were centrifuged at 3000 rpm for 15 min at 4°C, and then the supernatant was collected to prepare serum samples. The estrogen detection ELISA kit was used to determine the level of serum E2 (Shanghai Institute of Biological Product, Shanghai, China). All experimental steps referred to the product manual.

### 2.7. Autophagosome Observed by a Transmission Electron Microscope

The fresh NP tissue samples were immediately fixed in 1% glutaraldehyde for one day at room temperature and demineralized for 3 weeks. After that, the NP tissues were cut into 1 mm^3^. These samples were postfixed with 1% osmium tetroxide for 1 hour after washing with PBS. The treated samples were embedded in Durcupan ACM for 7 hours, cut into thin sections, which was stained with uranyl acetate and lead citrate, and finally examined with a Philips CM-80 transmission electron microscope (Eindhoven, Netherlands).

### 2.8. Western Blot Analysis

All the NP tissues were lysed in RIPA buffer (Beyotime, Jiangsu, China) containing PMSF and inhibitor of protease. The BCA kit (Beyotime, Jiangsu, China) was used to determine the concentration of protein in the tissue lysate according to the product manual. The tissue lysates (35 *μ*g) were separated by 12% SDS-PAGE using electrophoresis, and then the proteins were transferred to the nitrocellulose membrane. After blocking with 5% nonfat milk for 1 hour, the membranes were incubated with primary antibodies against LC3-B (1 : 1000, Novus, USA), Beclin1 (1 : 1000, Abcam, USA), p62 (1 : 1000, CST, USA), Atg5 (1 : 1000, CST, USA), and GAPDH (1 : 5000, Proteintech, China) at 4°C overnight. After being washed three times with PBS, the membranes were incubated with a LI-COR 800W secondary antibody (LI-COR Biosciences, USA). Finally, the membranes were determined by Odyssey machine (LI-COR Biosciences, USA) and were quantified by the IPP software.

### 2.9. Statistical Analyses

The present data were calculated and presented as means ± standard deviation (SD). One-way analysis of variance and *t*-test were used to analyze the significant difference by the SPSS 21.0 software. The correlation analysis was performed to correlate the serum estrogen level, autophagy level, status of redox balance, H-score, and the value of T2 mapping of IVD using Spearman's rank correlation coefficients. *P* < 0.05 demonstrated significant differences and *P* < 0.01 demonstrated highly significant differences.

## 3. Results

### 3.1. Verification of the Rat Lumbar IVDD

**Figure 2:**
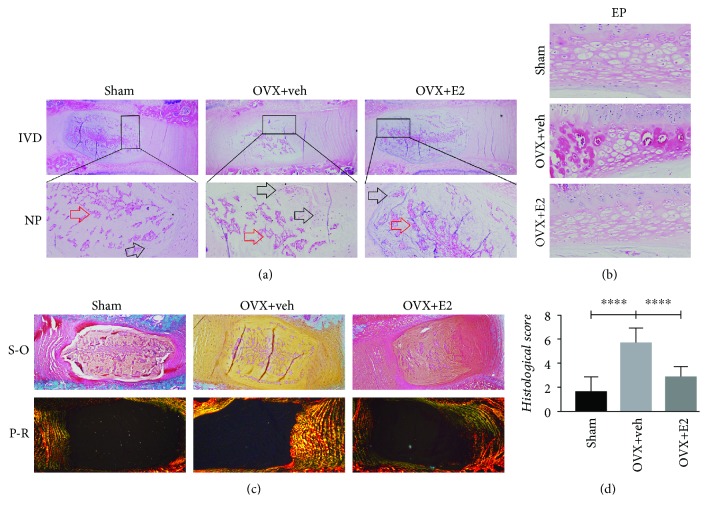
(a) Histological illustration of the L5–L6 IVDs 3 months postsurgery (original magnification, 4x). The arrows indicate the certain cell types in the NP tissue (red arrows: notochord cells; black arrows: chondrocyte-like cells; original magnification, 20x). (b) Ectopic bone tissue in EP. (c) Safranin-O (S-O) staining and picric acid-sirius red (P-R) staining of the IVD (original magnification, 4x). The red staining intensity for proteoglycan decreased in the NP and AF tissue with ovariectomy; estrogen supplementation can reverse the degeneration changes; collagen II was mainly displayed in the inner AF of the sham and OVX + E2 groups, but it was replaced by collagen I in the OVX group. (d) Histological score of disc degeneration of the three groups (^∗∗∗∗^*P* < 0.01 compared with the sham group and OVX + E2 group).

To investigate whether menopause plays a side role in causing IVDD, we established an ovariectomy rat model according to previous studies [15, 16]. Hematoxylin and eosin (HE) staining was used to assess the intervertebral disc (Figure 2(a)). In the OVX + veh group, the change of the cell phenotype occurred in the NP tissue. In the NP tissue, there were a significant number of notochord cells that are reduced, while many chondrocyte-like cells appeared in groups, and they were surrounded by the decrease in ECM in the NP tissue. The junction zone between AF and NP became blurring due to fissures, disorganized collagen fibers, and proliferation of fibrocartilage. However, the situation of the sham group was different because there were more notochord cells and a small number of cartilage-like cells. There was an intact and tightly arranging structure in the AF tissue of the sham group. The protective effects of estrogen were observed in the OVX + E2 group in which situations of the NP tissues were similar to those observed in the sham group.

The intervertebral discs were assessed using the scoring system. A good label was given to the sham group and its H-score was the lowest (1.6 ± 1.26). There were obvious pathological changes of the intervertebral disc in the OVX + veh group with a score of 5.7 ± 1.25, which was higher than the others (*P* < 0.05). Estrogen supplementation can partly alleviate the intervertebral disc degeneration; the score was 2.9 ± 0.88, which was lower than that of the OVX group (*P* < 0.05).

### 3.2. Estrogen Retards Degeneration of Disc Components

**Figure 3:**
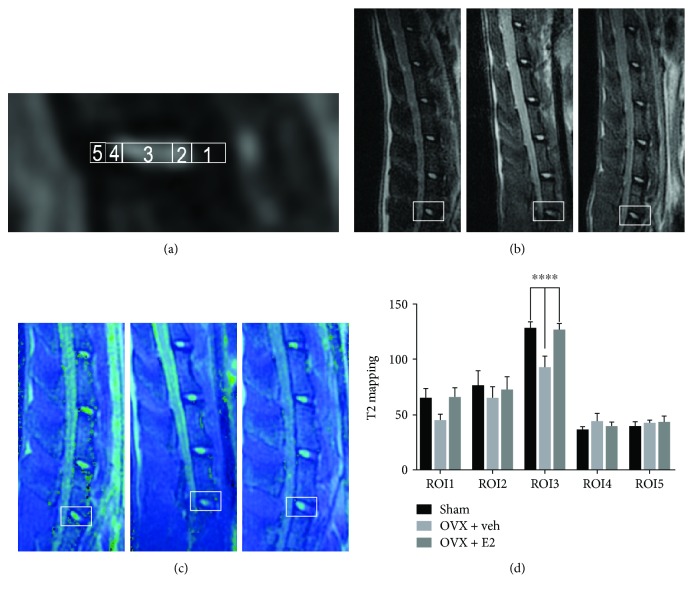
(a) Five regions of interest (ROIs) in the L5–L6 IVD were displayed. (b) MRI data of the discs were acquired at 3 months after surgery. (c) T2 mapping heatmap of the three groups was presented. (d) The values of T2 mapping (^∗∗∗∗^*P* < 0.01 compared with the sham group and OVX + E2 group).

**Figure 4:**
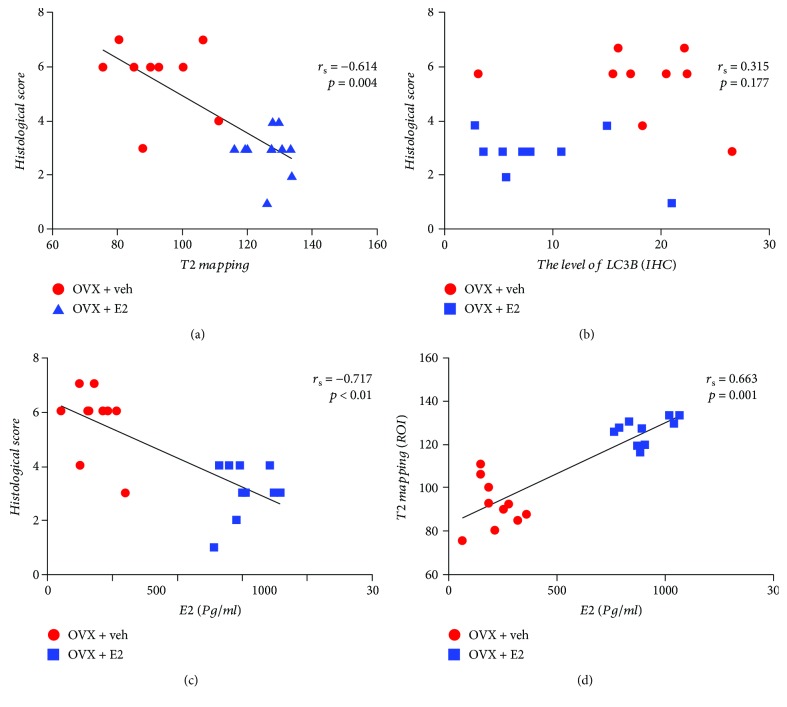
Spearman's correlation between the histological score and T2 mapping (a), level of LC3 (IHC) (b), and serum E2 level (c). Spearman's correlation between T2 mapping and serum E2 level.

To assess the protective effects of estrogen in vivo, estrogen supplementation was performed in the OVX + E2 group daily. The content of proteoglycan and collagen was determined by SO and PR staining. As shown in Figure 2(c), the decreased red staining intensity demonstrated the degradation of proteoglycan in the nucleus pulposus and annulus fibrosus tissues in the OVX rats, which indicated that estrogen supplementation can reverse the degradation. Polarization microscopy was used to determine the types of collagen, under which collagen type I fibrils exhibit strong yellow birefringence, while collagen type II fibrils exhibit slight multicolor birefringence. In the sham and OVX + E2 groups, the inner layer AF exhibited multicolor, and the outer layer AF showed strong density of yellow color, indicating that AF closest to the NP tissue was mainly collagen type II. However, the AF of the OVX group's discs presented yellow staining, which proves that their main component was composed of type I collagen (Figure 2(c)). Traditional methods of clinically evaluating IVD degeneration are typically performed using T2-weighted magnetic resonance imaging sequences. Regrettably, it failed to diagnose the early stage of degeneration of the intervertebral disc. A new kind of sequence, called T2 mapping, was believable to be a credible and sensitive tool to monitor the degradation of intervertebral disc content [26]. According to our data, the depression of the T2 mapping value is positively correlated with IVDD. The value acquired from T2 mapping of the NP (ROI3) decreased in the OVX groups compared with the sham group. Furthermore, such degradation can be reversed by drug intervention (Figure 3). The MR results have an inverse correlation with histological scores (Figure 4(a)).

### 3.3. Estrogen Supplementation Restored Redox Balance by Promoting Antioxidant Capacity

As shown in Table 1, the results of ELISA demonstrated that serum E2 level in OVX rats was significantly reduced (216.51 ± 88.08 pg/ml). Furthermore, the ovariectomy decreased the level of GSH (5.46 ± 1.33 U/mg prot) and increased the level of GSSG (4.92 ± 1.4 U/mg prot), which consequently increased the ratio of GSSG/GSH (1.30 ± 0.75). Moreover, ovariectomy resulted in a decrease in the capacity of T-AOC (1.69 ± 0.89 U/mg prot), SOD (22.65 ± 8.73 U/mg prot), and GSH-Px (34.47 ± 11.92 U/mg prot), while no changes were observed in the capacity of CAT (29.28 ± 10.86 U/mg prot). Estrogen supplementation in OVX rats made serum E2 level (906.14 ± 104.2 pg/ml) close to the sham group. The detection of redox balance showed a significant accumulation of oxidized glutathione (GSSG) and a consumption of reduced glutathione (GSH), suggesting that redox imbalance stress can be corrected by estrogen supplementation (Table 1). In the OVX + E2 group, the level of GSH (GSH 8.12 ± 1.33 U/mg prot) was higher and the GSSG (3.56 ± 0.87 U/mg prot) was lower than the OVX + veh group; as a consequence, the GSSG/GSH of the NP tissue was diminished by estrogen supplementation. Our data suggested that estrogen deprivation decreased the antioxidant capacity of the NP tissue. This imbalance of redox may be restored by estrogen replacement. The capacities of T-AOC (2.64 ± 0.92 U/mg prot), SOD (44.24 ± 13.74 U/mg prot), and GSH-Px (34.47 ± 11.92 U/mg prot) were enhanced by estrogen replacement. In contrast, CAT (30.08 ± 11.73 U/mg prot) capacity seemed not to be modulated by estrogen.

### 3.4. The Increasing Level of Autophagy in the OVX + veh Group

**Figure 5:**
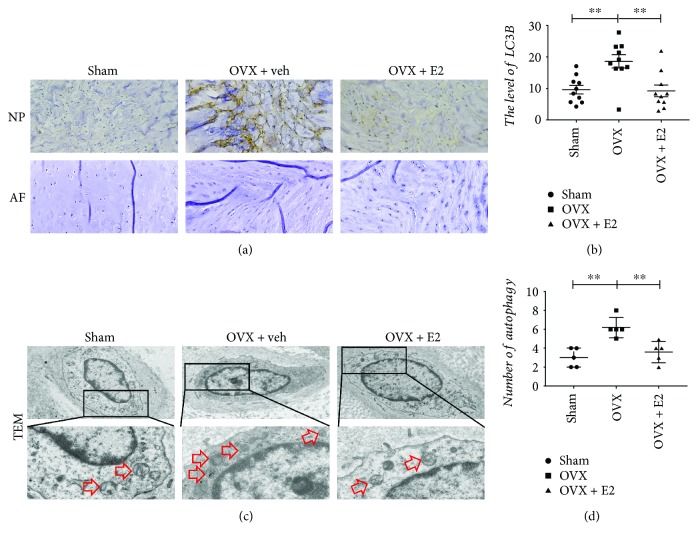
Autophagy in IVD assessed by IHC staining and TEM. (a) IHC staining for LC3 protein expression in the NP (original magnification, 20x). No significant difference in the AF. (b) Quantification of LC3 in the NP tissue, ^∗∗^*P* < 0.01. (c) The autophagosome was displayed in the cytoplasm of the NP cell as the red arrow showed among the three groups (original magnification, 2 K and 8 K). (d) Quantification of the number of autophagosomes per cell. Data were shown as mean ± SD (*n* = 5, ^∗∗^*P* < 0.01).

Autophagy plays as a protective effect when the NP cells are stimulated by inflammatory, compression stress, starvation, and/or oxidative stress. The hallmarkers of autophagy in the NP tissue, such as LC3-II/I, p62, Atg5, and Beclin1, were assessed by Western blot. Protein bolt results revealed that the protein level of Atg5, LC3, and Beclin1 increased in the OVX + veh group, while E2 therapy decreased the levels of Atg5, LC3, and Beclin1. However, the level of p62 had no difference compared with the other two groups (Figure 1). To further confirm that NP cells do produce the autophagy, immunohistochemistry was adopted. The similar trend of LC3 levels was determined compared with protein blot's result (Figure 5). Furthermore, TEM was used to detect the autophagosome of NP cells. As shown in Figure 5(d), NP cells of the OVX group possessed more autophagosomes than that of the other two groups. The present results indicated that there was a high level of autophagy in NP cells of the OVX group. In addition, there was no correlation between histological score and the level of LC3 (*r*_s_ = 0.315, *P* = 0.177) (Figure 4(b)).

### 3.5. Correlation between Serum E2 Levels and H-Score, Antioxidative Biomarkers, and Autophagy Levels in the NP Tissue

**Figure 6:**
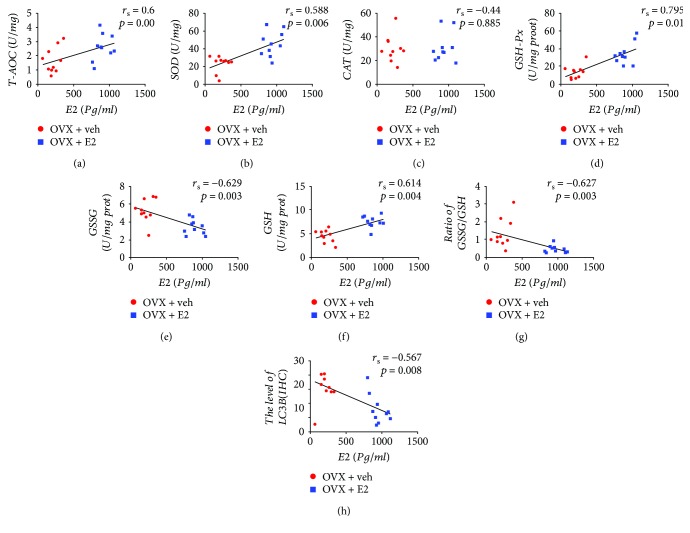
Spearman's correlation between serum E2 level and T-AOC (a), SOD (b), CAT (c), GSH-Px (d), GSSG (e), GSH (f), ratio of GSSG/GSH (g), and level of LC3 (IHC) (h).

To assess the relationship between E2 changes and redox balance, Spearman's correlation method was used to describe the correlation of serum E2 level with H-score, the value of T2 mapping, the capacity of T-AOC, SOD, CAT, GSH-Px, GSSG, GSH, and GSSG/GSH ratio, and autophagy levels in IVD of OVX rats and OVX + E2 rats. The results are reported in Figures 4 and 6. There was a significant correlation between the E2 and T2 mapping value (*r*_s_ = 0.663, *P* = 0.001), T-AOC (*r*_s_ = 0.6, *P* = 0.005), SOD (*r*_s_ = 0.558, *P* = 0.006), GSH-Px (*r*_s_ = 0.795, *P* < 0.01), and GSH (*r*_s_ = 0.614, *P* = 0.004); in contrast, changes in E2 were negatively correlated with H-score (*r*_s_ = −0.717, *P* < 0.01), GSSG (*r*_s_ = −0.629, *P* = 0.03), GSSG/GSH ratio (*r*_s_ = −0.627, *P* = 0.003), and LC3B (*r*_s_ = −0.576, *P* = 0.08). The changes in E2 level, however, cannot indicate variation of CAT capacity (*r*_s_ = 0.44, *P* = 0.885). In addition, there was a significant negative correlation between the LC3B level and T-AOC activity (*r*_s_ = −0.519, *P* = 0.019) and GSH (*r*_s_ = −0.45, *P* = 0.0.047). However, no significant correlation was found between LC3B level and SOD (*r*_s_ = −0.042, *P* = 0.079), CAT (*r*_s_ = 0.005, *P* = 0.985), GSH-Px (*r*_*s*_ = −0.042, *P* = 0.061), and GSSG (*r*_s_ = 0.361, *P* = 0.118).

## 4. Discussion

Due to the high prevalence of low back pain associated with IVDD in postmenopausal women and the limitation of treatment methods [2, 4, 6], it is important to find novel ways to retard IVDD and restore normal disc function. Many previous studies have demonstrated the effects of ERT on osteoporosis in postmenopausal women, but few reports focused on disc degeneration [2, 14]. Interestingly, our study showed that a negative correlation between degeneration of disc and level of estrogen was addressed in OVX rats. Low level of antioxidant status was correlated with degeneration of intervertebral disc and induced high autophagy level in the NP tissue. In addition, the imbalance in the redox status of disc can be corrected by ERT; meanwhile, the IVD degeneration can be reversed and autophagy can be downregulated by estrogen. Very interestingly, here we reported that ERT acted as a protective factor in the degeneration of intervertebral disc by modulating the redox balance and downregulating the autophagy level.

IVDD, caused by abnormal axial load and inappropriate internal environment of discs [27], is the major pathogenesis leading to low back pain, and higher prevalence of the pain associated with menopause has been reported in several literatures [25, 28, 29]. Evidences suggested that depression of estrogen level may be the main factor to IVDD in postmenopausal women [4, 5, 30]. However, there are few investigations to address the regulatory effect of estrogen on IVDD. In this study, we employed ovariectomy rat model to induce lumbar IVDD as performed in previous reports [15, 16] and found that OVX rats were susceptible to IVDD, in which the pathogenesis displayed in early stage of degeneration. The early stage of IVDD often starts with changing of cellular phenotype, degradation of extracellular matrix, and blurred border zone between AF and NP [8, 31–33]. In the OVX group, there was a reduced number of notochord cells and appearance of chondrocyte-like cells in the NP tissue, whereas estrogen supplementation can halt the above pathological process in the OVX + E2 group. Previous studies suggested that estrogen directly regulated the synthesis and function of collagen and proteoglycans [34]. In fact, IVDD contains various collagens and proteoglycans. The type II collagen, playing an important role in NP and the boundary between NP and AF, provides tensile strength to the disc [35]. The major proteoglycan of the NP tissue in IVD, aggrecan, played a major role in maintaining osmotic pressure and tissue hydration [36]. Here, the present study showed that IVDD, induced by low level of estrogen, started with the degradation of aggrecan and type II collagen in the OVX rats compared with the sham and OVX + E2 rats. Jia et al. [16] reported that the gene level of aggrecan and collagen II decreased in OVX rats, and at the same time, the ECM enzymes can be inhibited by the E2 treatment.

Oxidative stress, induced by redox imbalance, could cause damage to cellular and tissue components [37]. Recent studies have reported that the pathological morphology and development of IVDD are correlated with oxidative stress [18, 19], and oxidative stress targeted treatment may be a potential method to reverse the IVDD [38]. Importantly, estrogen depletion deranges redox balance in postmenopausal women [39], whereas ERT can decrease the lipid peroxides and promote antioxidant capacity [40]. Previous studies have reported that E2 promoted the capability to scavenge free radicals, suggesting that E2 could exert protective effect against oxidative damage by controlling the antioxidant signaling pathways [41, 42]. This study showed, for the first time, that the status of redox in IVD is closely managed by serum E2. Glutathione, a tripeptide and a major factor in cell survival, acts as cornerstone for intracellular antioxidant system [43]. There are two states of glutathione in cell and tissue, glutathione (GSH) and GSH disulfide (GSSG) [44]. The ratio of GSSG/GSH represents oxidative status of cell and tissue, and the normal ratio is critical for the cell to survival [45, 46]. Baeza et al. reported that OVX rats had a higher ratio of GSSG/GSH [47], and a recent report showed that depletion of estrogen induced high level of GSH-Px in OVX rats [48]. Otherwise, a series of antioxidant enzymes, including T-AOC, SOD, CAT, and GSH-Px, are the main tools to remove overproduction of active free radicals. Accumulating evidences about the effects of estrogen on the activities of these enzymes have been reported [20]. Kawvised et al. reported that, in hippocampus homogenates of OVX rats, the activities of SOD, CAT, and GSH-Px were reduced [48]. Recent studies reported the promotion effects of estrogen on antioxidant enzyme system [22, 40]. Estrogen promotes the expression of antioxidant enzymes system through activating MAPK and NF*κ*B pathways in vitro [49]. Another evidence provided on circulation of ovariectomized female showed that ERT increased antioxidant gene expression [22]. Our present results definitively supported such observations.

In addition to redox balance, Shen et al. [23] addressed that autophagy acted as a protective factor against oxidative stress through reducing oxidative stress. We, firstly, showed that autophagy levels increased in intervertebral disc in OVX rats. Our results suggested that autophagy might play a protective role to oxidative stress under hormone deprivation. Furthermore, we find that autophagy levels had inverse tendency compared with antioxidative biomarkers (T-AOC and GSH). Autophagy may protect cells by digesting oxidative damaged proteins and organelles [50]. Our results suggested that autophagy of the intervertebral disc is a response to the oxidative damage, and the levels of autophagy were decreased after that estrogen restored the redox balance.

There are some limitations that should be considered in this study. First, there was only one time point to investigate the effect of ERT on the redox balance in intervertebral disc. It may be better that the dynamic redox balance and treatment effects of ERT to IVDD should be evaluated under multitime points. Second, future studies could be included to confirm our conclusion, such as treating the OVX rats with antioxidative stress drugs to address whether IVDD can be retard by regulating the redox balance in IVD. Finally, the pathogenesis environment of IVD is different between rats and human; further studies should discuss the matter of human IVD.

**Figure 7:**
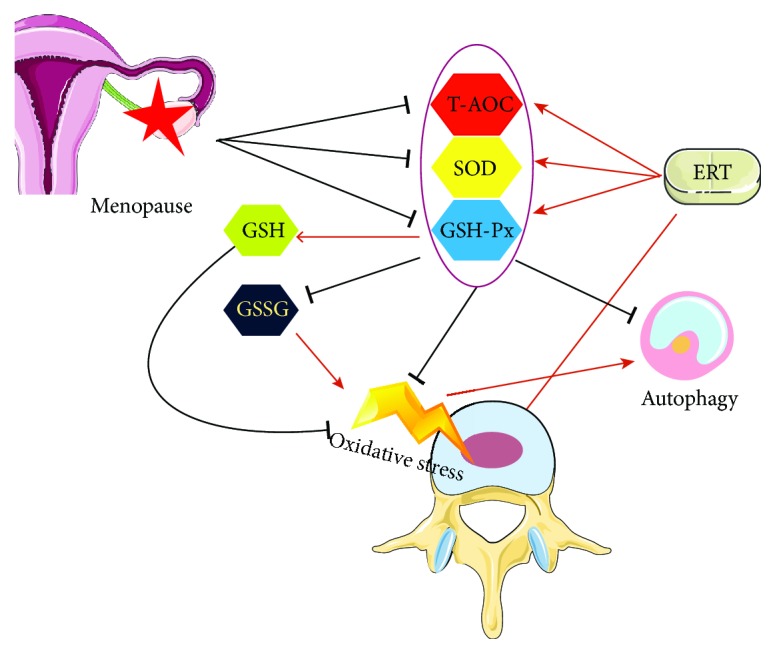
Schematic of the working hypothesis. Low level of estrogen leads to oxidative stress in the NP tissue of IVD, and at the same time, the level of autophagy increased as a response to the stress. ERT could modulate the redox balance, by which IVDD can be retarded partly.

In summary, the data demonstrated that disc redox balance was correlated with E2 levels; furthermore, IVDs in OVX rats were susceptible to degeneration due to estrogen deficiency. ERT was able to enhance the antioxidant capacity and reverse the degeneration of IVD, even though there were too few studies investigating the menopause-related intervertebral disc degeneration. These findings strongly suggested that estrogen may partly control antioxidant enzyme proteins' expression (Figure 7). Moreover, IVDD associated with menopause may depend on redox biology, and ERT may bring a positive effect for disc degeneration.

## Figures and Tables

**Table 1:** The level of E2 in serum and oxidative stress in IVD.

	Sham	OVX + veh	OVX + E2
Serum E2 (pg/ml)	949.18 ± 104.4	216.51 ± 88.08^∗∗^	906.14 ± 104.2^##^
T-AOC (units/mg protein)	3.88 ± 0.95	1.69 ± 0.89^∗∗^	2.64 ± 0.92^##^
CAT (units/mg protein)	28.73 ± 11.67	29.28 ± 10.86	30.08 ± 11.73
SOD (units/mg protein)	45.90 ± 12.29	22.65 ± 8.73^∗∗^	44.24 ± 13.74^##^
GSH-Px (units/mg protein)	35.67 ± 13.31	14.15 ± 7.46^∗∗^	34.47 ± 11.92^##^
GSSG (units/mg protein)	3.12 ± 1.13	5.46 ± 1.33^∗^	3.56 ± 0.87^#^
GSH (units/mg protein)	7.71 ± 2.26	4.92 ± 1.4^∗^	8.12 ± 1.33^#^
Ratio of GSSG/GSH	0.50 ± 0.24	1.30 ± 0.75^∗∗^	0.46 ± 0.19^##^

Data are expressed as mean ± SD. ^∗∗^*P* < 0.01, significant difference between OVX and sham. ^∗^*P* < 0.05, significant difference between OVX and sham. ^##^*P* < 0.01, significant difference between OVX and OVX + E2. ^#^*P* < 0.05, significant difference between OVX and OVX + E2.

## Data Availability

The data used to support the findings of this study are available from the corresponding author upon request.
